# Syndrome of Inappropriate Antidiuretic Hormone Secretion (SIADH) and Precocious Puberty With a Third Ventricle Arachnoid Cyst

**DOI:** 10.7759/cureus.22182

**Published:** 2022-02-13

**Authors:** Ahmad Imam, Abdullah A Tawakul, Khalil F Miyajan, Zaid A Majeed, Colleen Buggs-Saxton

**Affiliations:** 1 Internal Medicine / Endocrinology, Umm Al Qura University, Makkah, SAU; 2 Department of Internal Medicine / Neurology, Umm Al-Qura University, Makkah, SAU; 3 Faculty of Medicine, Umm Al Qura University, Makkah, SAU; 4 Faculty of Medicine, Umm Al-Qura University, Makkah, SAU; 5 Pediatric Endocrinology, Wayne State University School of Medicine, Detroit, USA

**Keywords:** third ventricle, syndrome of inappropriate antidiuretic hormone, precocious puberty, growth hormone deficiency, arachnoid cyst

## Abstract

Arachnoid cyst (AC) is a rare defect of the central nervous system that accounts for 1% of all intracranial lesions, of which only 1% of reported cases are located in the third ventricle. Endocrine manifestations associated with AC include precocious puberty, growth hormone deficiency, and hypothalamic dysfunction. We report a child who presented with a visual field defect, hyponatremia, and precocious puberty related to a third ventricle AC. Hyponatremia as a complication of AC is rare. A literature review revealed two case reports of Syndrome of inappropriate antidiuretic hormone secretion (SIADH) associated with suprasellar AC. The pathophysiology of SIADH in AC is not well understood. Hyponatremia may worsen following endoscopic fenestration of the AC secondary to changes in intracranial pressure. In conclusion, hyponatremia with AC should be recognized during the preoperative and postoperative periods and may require treatment with hypertonic saline in addition to fluid restriction.

## Introduction

Arachnoid cysts (AC) are non-cancerous cysts that contain cerebrospinal fluid (CSF) [1]. It is a rare defect of the central nervous system in the cerebrospinal axis in relation to the arachnoid membrane that does not communicate with the ventricular system. It accounts for 1% of all intracranial lesions, of which only 1% of reported cases are located in the third ventricle [2,3]. Although the mechanism of progressive enlargement of the cyst over time is not well understood, it could be due to passive CSF diffusion into the cyst or by the ball-valve effect [4,5]. The original defect of the AC could be due to a congenital malformation of the neural tube, head trauma, or prior intracranial procedure [1].

Most of these lesions are found incidentally while undergoing neuroimaging for other unrelated problems. Moreover, the majority of AC patients are asymptomatic while some may present with headaches, increased head circumference, and developmental delay in pediatric patients. Rarely, due to raised intracranial pressure, the patients may develop symptoms of weakness, seizures, and endocrinopathies, depending on the location of the AC [1]. Endocrine manifestations of AC are exceptionally rare and may include growth hormone (GH) deficiency, hypothalamic dysfunction, and precocious puberty [6]. Morbidity and mortality are dependent on the location of the AC and the presence of complications such as intracystic bleeding or the formation of a subdural hygroma or hematoma [7]. To our knowledge, hyponatremia was not described as a feature of a suprasellar arachnoid cyst until recently. We describe a child who presented with a visual field defect, hyponatremia, and precocious puberty related to a third ventricle AC.

## Case presentation

A 9-year-old boy presented with decreased vision in the left eye for eight months. He denied any headaches, nausea, or vomiting. His past medical history was unremarkable. On physical examination, his vital signs were stable; height and BMI were at the 90th percentile and weight was at the 94th percentile. He was alert, conscious, and oriented to time, place, and person. Visual exam through Snellen’s chart indicated a loss of vision and impaired visual field in the left eye. He had normal sensory, motor, and reflexes exam of the upper and lower extremities. Pubic and axillary hairs were at the Tanner I stage, testicles were 3 cm bilaterally (Tanner II), and penile length was 7.5 cm.

Laboratory tests (Table 1) revealed hyponatremia 132 mMol/L, low serum osmolality 275 mOsm/kg, an inappropriately elevated urine osmolality 886 mOsm/kg, pubertal testosterone 109 ng/dL, a pubertal luteinizing hormone level (LH) 2.0 mIU/mL and follicle-stimulating hormone (FSH) 5 mIU/mL, an advanced bone age (12 years); normal insulin-like growth factor binding protein 3 (IGFBP3) 3.5 mcg/mL, normal thyroid-stimulating hormone (TSH) 0.89 uIU/mL, and free thyroxine (FT4) 0.92 ng/dL.

**Table 1:** Patient’s laboratory tests mMol/L: Millimoles per liter, mOsm/kg: Milliosmoles per kilogram, ng/dL: Nanograms per deciliter, mIU/mL: Milli-international units per milliliter, uIU/mL: Micro-international units per milliliter, pg/mL: Picograms per milliliter, mcg/dL: Micrograms per deciliter.

Test	Result	Reference range
Sodium	132 mMol/L	135 – 145 mMol/L
Serum osmolarity	275 mOsm/kg	280–295 mOsm/kg
Urine osmolality	886 mOsm/kg	50-1200 mOsm/kg
Pubertal testosterone	109 ng/dL	1.80 – 5.68 ng/dL
Luteinizing hormone (LH)	2.0 mIU/mL	<0.02-3.6 mIU/mL
Follicle-stimulating hormone (FSH)	5 mIU/mL	≤2.3 mIU/mL
Adrenocorticotropic hormone (ACTH)	15 pg/mL	5-46 pg/mL
Serum AM cortisol	8.2 mcg/dL	3-30 mcg/dL
Insulin-like growth factor binding protein 3 (IGFBP3)	3.5 mcg/mL	2.3-6.3 mcg/mL
Thyroid-stimulating hormone (TSH)	0.89 uIU/mL	0.34-4.8 uIU/mL
Free thyroxine (T4)	0.92 ng/dL	0.6-1.8 ng/dL
Cortisol response to 1 mcg cosyntropin	19.4 mcg/dL	>18 mcg/dL

**Figure 1:**
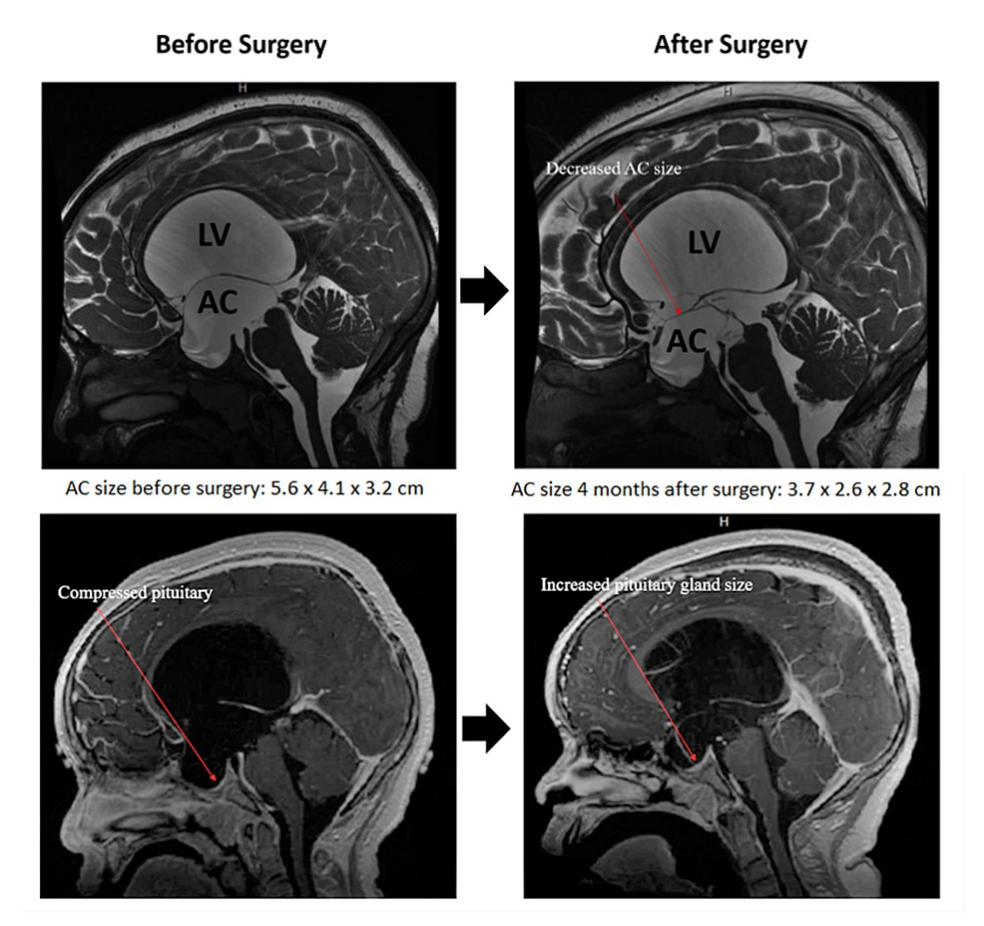
MRI brain+/-contrast showing the arachnoid cyst (AC), enlarged lateral ventricle (LV), and compressed pituitary gland. Postoperative change is illustrated.

A brain MRI was ordered for further evaluation of central precocious puberty and hyponatremia. The initial brain MRI with contrast demonstrated a 5.6x4.1x3.2 cm third ventricle AC, severe obstructive hydrocephalus, enlarged sella with a mass effect on the pituitary gland, and deviation of the pituitary stalk along with papilledema (Figure 1).

The patient was immediately admitted for endoscopic fenestration of the AC the next day. He was diagnosed with a third ventricle AC, causing syndrome of inappropriate antidiuretic hormone secretion (SIADH). Prior to surgery, his hyponatremia worsened (Na 129 mMol/L) and the patient had a significant headache. Treatment with 3% hypertonic saline was given for symptomatic hyponatremia and to prevent further worsening of hyponatremia during surgery. Postoperatively, hyponatremia improved (Na 134 mMol/L) and he was kept on 1200 ml per 24-hour fluid restriction. Three days after discharge, he was readmitted with a history of altered mental status and worsening hyponatremia (Na 128 mMol/L) requiring treatment with 3% hypertonic saline. A low dose ACTH-stimulation test (1 mcg) was performed to rule out adrenal deficiency and revealed normal cortisol response (19.4 mcg/dL). His sodium improved gradually and reached 132 mMol/L (Table 2). He was then discharged with instructions to continue 1200 ml fluid restriction and increased dietary salt intake (2 grams daily). Four months after the surgery, his sodium level was 133 mMol/L. A repeat MRI at that time showed decreased size of the SACs (from 5.6 x 4.1 x 3.2 cm to 3.7 x 2.6 x 2.8 cm) and improvement in the associated mass effect on the surrounding structures (Figure 1).

**Table 2:** Sodium level summary and intervention: mMol/L: Millimoles per liter, mL: Milliliter

	Before surgery	24-hour after surgery	3 days after surgery	On discharge	4 days after discharge (readmission)	4 months later (outpatient)
Serum sodium (mMol/L)	129	135	127	130	128	133
Symptoms	Yes	No	No	No	Yes	No
Intervention	3% hypertonic saline	-	1200 mL fluid restriction	1200 mL fluid restriction	3% hypertonic saline	1200 mL fluid restriction
	Endoscopic fenestration of the AC	-	-	-	1200 mL fluid restriction	Increase dietary salt
	-	-	-	-	Increase dietary salt	-

## Discussion

Suprasellar arachnoid cysts (SACs) are rare; they represent 5-12% of intracranial arachnoid cysts [8]. The actual mechanism behind AC formation is not well understood and pathological features of the cyst tend to follow its location [9]. Based on the literature, the patient with a SAC typically presents with an enlarged skull, obstructive hydrocephalus, slowing down of psychomotor movements, and prenatal discovery of the cyst incidentally. Additionally, due to mass effect, the patient may also present with headaches, nausea and vomiting, seizures, decreased visual acuity, endocrine disorders, headbobbing, neurological deficits, and a wide variety of endocrine manifestations [10-13]. SACs can lead to hormonal disturbances as they are located near the hypothalamic-pituitary area [14]. The most common reported endocrine manifestation of SACs is precocious puberty which is suggested to be related to the disruption of posterior hypothalamus inhibitory signals on the gonadotrophs [15].

SIADH with SACs is not commonly reported. Our literature review revealed only two case reports of SIADH associated with SAC [16,17]. The pathophysiology of SIADH in SAC is not well understood, but one proposed mechanism suggests that the SAC may directly stimulate arginine vasopressin (AVP) neurons in the paraventricular and supraoptic nuclei in the hypothalamus [18]. Regarding our patient, the suggested mechanism for SIADH is the compression of the supraoptic and paraventricular nuclei by the enlarged SAC, which overstimulated the secretion of AVP, resulting in SIADH [19].

Typically, imaging is used to make the diagnosis of SACs as they are well-defined cysts with a thin wall that displace surrounding structures that follow CSF density on CT and CSF signal intensity on MRI [1]. In our patient, the MRI showed AC with an enlarged lateral ventricle (LV) and third ventricle that compressed the pituitary gland so that our patient was immediately admitted for endoscopic fenestration of the AC the next day.

Treatment is usually required for symptomatic patients. If the lesion is asymptomatic, serial imaging and neurologic exams are required to follow up [6]. According to the available literature, decompressive surgery is indicated when symptoms of increased intracranial pressure, seizures, focal neurologic deficits, cognitive impairment, or other endocrinal manifestations develop. Surgical treatment options include craniotomy for complete or partial cystectomy, fenestration into the subarachnoid space, and cyst peritoneal shunting [1,6]. In special cases when SACs present with hyponatremia, fluid management is important especially during the pre- and postoperative periods and may require treatment with hypertonic saline in addition to fluid restriction [19].

However, even after successful decompression, hyponatremia may worsen following the endoscopic fenestration of the AC secondary to changes in intracranial pressure [20]. In our patient, the hyponatremia deteriorated before surgery (sodium 129 mmol/L, requiring treatment with 3% hypertonic saline solution). Then, the patient underwent a successful endoscopic fenestration without complications. After surgery, the hyponatremia improved (Na 134 mmol/L) with fluid restriction and increased dietary salt intake due to the short duration of this compression. 

## Conclusions

SACs associated with SIADH in children are extremely rare and can cause other endocrinologic complications, such as precocious puberty. Additionally, the immediate cyst fenestration could improve the patient's SIADH, as observed in the case above. Moreover, it is important to consider SIADH as a cause of hyponatremia in pediatric patients with SACs.
